# Tissue-specific oxidative responses and recovery following acute thermal stress in juvenile Amazonian fish *Colossoma macropomum*

**DOI:** 10.1007/s10695-026-01766-1

**Published:** 2026-08-04

**Authors:** Naiara Melo, Isaac Konig, Eduarda Rodrigues Barbosa, Stefania Priscilla de Souza, Sidney dos Santos Silva, Ronald Kennedy Luz, Priscila Vieira Rosa, Luis David Solis Murgas

**Affiliations:** 1 Faculty of Animal Science and Veterinary Medicine, Federal University of Lavras, UFLA, Lavras, Minas Gerais Brazil; 2 Department of Biochemistry, Federal University of Rio Grande Do Sul, UFRGS, Porto Alegre, Rio Grande Do Sul Brazil; 3 Departamento de Zootecnia, Laboratório de Aquacultura, Universidade Federal de Minas Gerais, Avenida Antônio Carlos, 6627, Belo Horizonte, Minas Gerais zip code 30161-970 Brazil

**Keywords:** Tambaqui, Acute thermal stress, Oxidative stress, Antioxidant enzymes, Integrated biomarker response (IBRv2)

## Abstract

*Colossoma macropomum* (tambaqui) is an economically important Amazonian fish increasingly exposed to heatwaves in tropical aquaculture systems. However, multi-tissue responses to acute thermal stress and recovery remain poorly understood. Juveniles were exposed to 34 °C for 24 h, with samples collected at baseline and at 1, 24, and 48 h post-recovery (28 °C). Antioxidant responses and oxidative damage markers were evaluated to determine whether tambaqui re-establishes physiological homeostasis following acute heat shock. Antioxidant enzymes showed significant time- and tissue-dependent changes following acute heat shock. Overall, SOD, CAT, GPx, GR, and GST activities were transiently altered during the early recovery phase (1–24 h), but most enzymes returned to baseline levels within 48 h in the brain, gills, intestine (except CAT), liver, and muscle (except SOD) (p > 0.05). Regarding oxidative damage markers (PC and MDA), the intestine was the most affected tissue, with significantly higher levels at 48 h than at baseline. Based on integrated biomarker analysis (IBRv2), gills and muscle showed the smallest deviation from baseline levels after 48 h, indicating a more complete recovery compared to the other tissues. The intestine, on the other hand, showed persistent oxidative damage markers even at 48 h of recovery. Overall, the results indicate that although acute heat shock induces transient, tissue-specific oxidative disturbances, juvenile tambaqui largely restore redox homeostasis within 48 h, with recovery capacity varying among tissues.

**Supplementary Information:**

The online version contains supplementary material available at https://doi.org/10.1007/s10695-026-01766-1.

## Introduction

*Colossoma macropomum* (Cuvier, 1818) (Characiformes: Serrasalmidae), popularly known as tambaqui, is a Neotropical fish native to the Amazon and Orinoco river basins and one of the main species produced by inland aquaculture in South America. It is also farmed in Asian countries (Valladão et al. 2018; Hilsdorf et al. 2022). The species’ productive relevance stems from its rapid growth under tropical conditions, its hardiness in the face of management challenges (e.g., high stocking densities and low oxygen availability), and its efficient utilization of formulated feeds (Paulino et al. 2018; Ananias et al. 2024; Melo et al. 2024b; Mattioli et al. 2025). In addition to its productive importance, tambaqui is associated with Amazonian floodplains, where the flood pulse (Junk et al. 1989) exposes it to seasonal fluctuations in habitat and water quality (Brito et al. 2013), reinforcing its physiological plasticity in response to abiotic stressors (Jardine et al. 2025; de Oliveira et al. 2026).

Among abiotic factors, temperature is a key environmental variable for fish, as it regulates the kinetics of biochemical reactions and, due to ectothermy, constrains energy balance and processes such as feeding, digestion, and growth (Volkoff and Rønnestad 2020; Mugwanya et al. 2022). Thus, abrupt thermal fluctuations may disrupt physiological homeostasis and increase metabolic maintenance costs. This effect is particularly pronounced when the change occurs over a short time interval and exceeds the organism’s capacity for adjustment (Volkoff and Rønnestad 2020; Abdel-Tawwab et al. 2025; Abdul Kari 2025). In the context of climate change, heatwaves in rivers have intensified and are expected to become more persistent, increasing the likelihood of acute heat exposure for aquatic organisms, particularly in tropical regions (Sadayappan and Li 2025; Chen et al. 2026). Such conditions increase the probability of acute heat exposure in both natural ecosystems and different aquaculture production systems, for which heatwaves are already considered an emerging challenge and are often associated with concurrent changes in water quality (Mugwanya et al. 2022; Riesco et al. 2026).

In this context, alterations in redox balance associated with climate change, particularly thermal fluctuations and rapid warming events or heatwaves, have been extensively studied in fish. Temperature increases may elevate the production of reactive oxygen species (ROS) and require adjustments of the antioxidant system, reflected in the modulation of antioxidant enzymes and oxidative damage biomarkers (Ritchie and Friesen 2022; Loughland et al. 2022). Such responses have been described in teleosts from different habitats subjected to acute or prolonged heat exposure (Vinagre et al. 2012; Ibrahim et al. 2019; Feidantsis et al. 2020; Loughland et al. 2022; Dawood et al. 2022; Hosseinpour et al. 2024; Perry et al. 2024; Sun et al. 2025). In *C*. *macropomum*, recent evidence indicates that warming, alone or combined with additional variables such as photoperiod, pH, and hypoxia, may also alter antioxidant responses and tissue damage markers, with implications for health and reproduction (Castro et al. 2020; Pereira et al. 2021). Moreover, prolonged exposure to thermal gradients reinforces 34 °C as an informative sublethal threshold for investigating heat responses in this species (Amanajás et al. 2024).

Despite this progress, the interpretation of redox and damage biomarkers is strongly tissue-dependent, as organs differ in function and metabolic demand. Therefore, multi-tissue approaches may reveal contrasting patterns among liver, intestine, gills, muscle, and plasma under different stressors (Dawood et al. 2022; Melo et al. 2024a). For tambaqui, although there is evidence of antioxidant and damage alterations under prolonged warming and under thermal shock in a single tissue (liver under cold shock) (dos Santos Silva et al. 2024; Amanajás et al. 2024), studies integrating multiple tissues within the same experimental protocol under acute heat shock remain limited. Although Amanajás et al. (2024) identified sublethal thermal thresholds and measured liver responses under acute heat shock, they assessed only a limited set of antioxidant enzymes and lipid peroxidation (LPO), highlighting the need for a multi-tissue approach and the evaluation of additional oxidative stress and antioxidant markers to better elucidate the physiological responses across tissues following thermal stress.

Therefore, the present study aimed to evaluate antioxidant responses and oxidative damage markers in different tissues following acute thermal shock at 34 °C. We measured the activity of enzymes related to oxidative stress, as well as tissue damage biomarkers (protein carbonyls, PC, and malondialdehyde, MDA), in multiple tissues over time after heat exposure. By adopting a multi-tissue and time-course approach, this study seeks to refine the understanding of the physiological costs of rapid warming and to determine whether tambaqui are able to re-establish physiological homeostasis after acute heat shock. The findings of this study are expected to generate information applicable to the management of the species in natural environments and aquaculture systems.

## Materials and methods

### Animal source and maintenance conditions

The experiment was conducted at the Department of Animal Science of the Federal University of Lavras (UFLA), Lavras, Minas Gerais, Brazil. All experimental procedures and sample collections were approved by the Animal Ethics Committee (CEUA) of UFLA under protocol no. 29/2017.

Juvenile *C*. *macropomum* were obtained from stocks maintained at the Aquaculture Laboratory (LAQUA) of the Federal University of Minas Gerais (UFMG, Brazil), with an initial body weight of 74.78 ± 6.66 g and total length of 17.10 ± 0.97 cm. Fish were randomly assigned to three rectangular tanks (100 × 69 × 58 cm) with a working volume of 400 L, at a stocking density of 24 fish per tank. The tanks were connected to a temperature-controlled recirculating aquaculture system (RAS) equipped with aeration, mechanical and biological filtration, and ultraviolet sterilization. The photoperiod was set at 12 h light:12 h dark (timer Brasfort 8769). Water quality parameters were maintained as follows: temperature 28.12 ± 0.25 °C, dissolved oxygen 6.91 ± 0.27 mg L⁻^1^, and pH 6.65 ± 0.43, measured using a multiparameter probe (AK88 + multiparameter meter). Total ammonia, measured daily using a colorimetric kit (Alfakit®), was kept below 0.3 mg L⁻^1^. During the 15-day acclimation period, fish were fed three times daily (08:00, 12:00, and 16:00 h) with a commercial extruded diet (Aquos tropical; 2–4 mm pellet size) containing 320 g kg⁻^1^ crude protein, 7.5 g kg⁻^1^ lipids, 120 g kg⁻^1^ ash, and 4.5 g kg⁻^1^ fiber.

### Experimental conditions

After the acclimation period, fish were fasted for 24 h to standardize the metabolic state. Acute thermal shock was induced by a controlled increase in water temperature in the RAS from 28 °C to 34 °C. Heating was achieved using a 400 L auxiliary reservoir, initially isolated from the RAS and preheated to 48 °C using a 2000 W portable immersion water heater. To increase the water temperature, the outlet of the RAS containing water at 28 °C was partially opened, allowing part of the cooler water to be removed while the preheated water from the auxiliary reservoir entered the system and mixed with the recirculating water. During the heating phase, water temperature was recorded every minute until the target temperature was reached. The target temperature was reached in approximately 32 min, corresponding to an average warming rate of 11.25 °C h⁻^1^. Simultaneously, the RAS temperature controller was set to 34 °C. After reaching the target temperature, water temperature was monitored hourly throughout the 24 h thermal stress period. Fish were maintained at 34.07 ± 0.27 °C for 24 h. After the 24 h exposure at 34 °C, the RAS temperature controller set point was decreased stepwise by 1 °C every hour until the water temperature returned to 28 °C. Recovery time was counted from the moment 28 °C was re-established (Time 0).

### Sampling

Sampling was performed at baseline (before temperature elevation) and at 1 h, 24 h, and 48 h after the return to 28 °C (end of heat stress). At each sampling point, six fish were collected from each tank, totaling 18 fish per time point. Following the methodology described by Melo et al. (2024a), fish were first anesthetized with eugenol (25 µL L⁻^1^) and subsequently euthanized by medullary section. After which the brain, gills, intestine, liver, and a portion of white muscle from the dorsal region were excised. Tissues were immediately frozen in liquid nitrogen and stored at − 80 °C until further processing. For statistical purposes, fish sampled from the same tank at the same time point were considered biological subsamples. Individual biomarker values were averaged within each tank, and the resulting tank mean was used as the statistical unit.

### Assessment of enzymatic responses

Tissues were first weighed on an analytical balance (Shimadzu AUW220D) and then homogenized in ice-cold saline buffer (0.138 M NaCl; 0.0027 M KCl; pH 7.2) at a 1:10 (w/v) dilution. The homogenates were centrifuged at 7,000 × g for 15 min at 4 °C (Multifuge X1R Centrifuge, Thermo Fisher Scientific, Waltham, MA, USA). The resulting supernatant was carefully collected, split into four 500 μL aliquots, and stored at − 80 °C until further analysis. All determinations were performed in technical triplicate using a microplate reader. The time between sample collection and completion of the assays was approximately one week.

#### Superoxide dismutase activity

Superoxide dismutase (SOD; EC 1.15.1.1) activity was determined using a modified protocol based on Madesh and Balasubramanian (1997). Briefly, 40 μL of homogenate were combined with 132 μL of PBS (Sigma-Aldrich, St. Louis, MO, USA) in a microplate. Then, 8 μL of 0.6 g/L MTT [3-(4,5-dimethylthiazol-2-yl)−2,5-diphenyltetrazolium bromide] and 20 μL of 1.25 mM pyrogallol (both from Sigma-Aldrich, St. Louis, MO, USA) were added. The reaction mixture was incubated at 37 °C for 5 min. After incubation, 150 μL of DMSO (Sigma-Aldrich, St. Louis, MO, USA) was added, and absorbance was recorded at 570 nm in triplicate using a microplate reader (Multiskan GO, Thermo Fisher Scientific, Waltham, MA, USA). Negative controls contained all reagents except pyrogallol and the sample. Total SOD activity was expressed as units (U) per milligram of protein, where one unit corresponds to the amount of enzyme required to inhibit 50% of superoxide radical dismutation per minute.

#### Catalase activity

Catalase (CAT; EC 1.11.1.6) activity was quantified according to a protocol adapted from Aebi (1984), which is based on monitoring the decomposition of hydrogen peroxide (H₂O₂). Initially, 10 μL of the supernatant was mixed with 90 μL of PBS to obtain a 1:10 dilution. Subsequently, 10 μL of the diluted sample was added to 140 μL of 10 mM H₂O₂ (Sigma-Aldrich, St. Louis, MO, USA) in a microplate well. The decrease in absorbance was recorded at 240 nm at 10-s intervals for two minutes, with all measurements performed in triplicate. CAT activity was expressed as units (U) per milligram of protein.

#### Glutathione peroxidase activity

Glutathione peroxidase (GPx; EC 1.11.1.9) activity was assessed following the procedure described by Flohé and Günzler (1984). The assay medium was prepared with 10 mL of PBS, 2.6 mL of 10 mM reduced glutathione (GSH), 2.6 mL of 1.6 mM NADPH, 2.6 mL of 10 mM sodium azide (NaN₃), and 1 μL of glutathione reductase (GR; 0.5 U mL⁻^1^). For the analysis, 40 μL of homogenate were dispensed into each microplate well, followed by 200 μL of the reaction medium and 25 μL of 10 mM hydrogen peroxide to initiate the reaction. The decrease in absorbance was recorded at 340 nm, and GPx activity was calculated and expressed as units (U) per milligram of protein.

#### Glutathione reductase activity

Glutathione reductase (GR; EC 1.8.1.7) activity was assessed according to Carlberg and Mannervik (1975). Briefly, 25 µL of homogenate were added to a 96-well microplate containing 200 µL of reaction mixture composed of 8 mL PBS buffer, 2 mL GSSG solution, 2 mL NADPH solution, 4 mL DTPA (diethylenetriaminepentaacetic acid) solution, and 6 mL deionized water. Enzymatic activity was monitored kinetically at 340 nm, based on NADPH oxidation. GR activity was calculated and expressed as units (U) per milligram of protein.

#### Glutathione S-transferase activity

Glutathione S-transferase (GST; EC 2.5.1.18) activity was determined based on the procedure reported by Habig et al. (1974), which employs 1-chloro-2,4-dinitrobenzene (CDNB) and GSH as substrates. For the assay, 25 μL of homogenate were placed in each microplate well and mixed with 125 μL of reaction solution containing 380 μL of 0.1 M CDNB in ethanol, 2.3 mL of GSH (30.7 mg mL⁻^1^), and 12.32 mL of PBS. The formation of the conjugate was monitored by measuring absorbance at 340 nm every 20 s, totaling 15 readings, with analyses performed in triplicate using a microplate reader. Wells containing only PBS served as the negative control. GST activity was expressed as units (U) per milligram of protein.

### Assessment of tissue damage biomarkers

#### Lipid peroxidation

Lipid peroxidation was determined by the TBARS method according to Buege and Aust (1978). For the assay, 300 µL of the homogenate (Sect. 2.4) was mixed with 200 µL of the TBARS solution. The TBARS solution was prepared by dissolving 30 g of trichloroacetic acid (TCA) and 0.75 g of thiobarbituric acid (TBA) in 100 mL of deionized water, then adding 12.5 mL of 4 M HCl. The final volume was adjusted with an additional 100 mL of deionized water and heated in a water bath at 60 °C until complete dissolution. Then, the mixture (homogenate and TBARS solution) was vortexed and incubated in a water bath at 37 °C for 15 min. After cooling to room temperature, 420 µL of butyl alcohol was added to the solution, which was again vortexed and centrifuged for 5 min at 5000 g. The supernatant from all samples was read in 96-well flat-bottom microplates at 532 nm. MDA concentrations obtained from the standard curve were normalized to total protein content, and lipid peroxidation results were expressed as nmol MDA mg protein⁻.^1^

#### Protein carbonyl levels

Protein carbonyl (PC) content was determined by 2,4-dinitrophenylhydrazine (DNPH) derivatization (Reznick and Packer 1994). Briefly, 200 µL of each sample was divided into two tubes (sample and control). DNPH (800 µL) was added to the sample tube, and 2.5 M HCl (800 µL) was added to the control. After incubation in the dark for 1 h at room temperature, proteins were precipitated with 20% TCA and centrifuged (10,000 × g, 10 min, 4 °C). Pellets were washed with 10% TCA and three times with ethanol/ethyl acetate (1:1), followed by resuspension in 500 µL of guanidine hydrochloride and centrifugation. Supernatants were transferred in triplicate to a 96-well plate, and absorbance was read at 370 nm. Results were calculated from the difference between the sample and control and expressed relative to total protein content. Protein carbonyl content was calculated using an extinction coefficient of 0.011 µM⁻.^1^

#### Quantification of protein content

Protein levels in the homogenates were determined according to Bradford (1976). Briefly, 10 µL of the supernatant was combined with 200 µL of Bradford reagent. A standard calibration curve was prepared using albumin. Absorbance readings were taken at 595 nm in triplicate. The resulting protein concentrations were subsequently used to normalize the enzymatic assay data described in Sect. 2.4 and the tissue damage biomarkers in Sect. 2.5.

### Integrated Biomarker Response version 2 (IBRv2)

The IBRv2 index was calculated as described by Sanchez et al. (2013). For each tissue, biomarkers were measured at baseline (0 h) and at 1, 24, and 48 h after thermal stress. For each biomarker i and time point t, values were aggregated as the mean per time point:

$${X}_{i,t}=\frac{1}{n}\sum_{k=1}^{n}{x}_{i,t,k}$$

where ${X}_{i,t}$ is the mean value of the biomarker i at time t, ${x}_{i,t,k}$ is the value measured in replicate k, and n is the number of tank-level replicates.

Each biomarker i was normalized relative to baseline using a logarithmic transformation:

$${Y}_{i,t}=\mathrm{l}\mathrm{n}\left(\frac{{X}_{i,t}}{{X}_{i,0}}\right)$$

where ${Y}_{i,t}$ is the normalized value of the biomarker i at time t, ${X}_{i,0}$ is the baseline value at 0 h, and $\mathrm{l}\mathrm{n}$ denotes the natural logarithm.

Subsequently, ${Y}_{i,t}$ was standardized for each biomarker:

$${Z}_{i,t}=\frac{{Y}_{i,t}-\overline{{Y}_{i}}}{S{D}_{i}}$$

where ${Z}_{i,t}$ is the standardized value of biomarker i at time t, $\overline{{Y}_{i}}$ is the mean normalized value of biomarker i across all time points, and $S{D}_{i}$ is the corresponding standard deviation.

The deviation relative to the control was calculated as follows:

$${A}_{i,t}={Z}_{i,t}-{Z}_{i,0}$$

where ${A}_{i,t}$ represents the deviation of biomarker i at time t relative to the baseline/control condition, and ${Z}_{i,0}$ is the standardized value at 0 h.

The IBRv2 index at each time point was obtained by:

$$IBRv{2}_{t}=\sum_{i=1}^{m}\mid {A}_{i,t}\mid$$

where m is the number of biomarkers included in the analysis.

The ΣIBRv2 value for each tissue was defined as the sum of the indices throughout the experimental period:

$$\Sigma IBRv2=IBRv{2}_{1h}+IBRv{2}_{24h}+IBRv{2}_{48h}$$

Calculations were performed in Microsoft Excel, and radar plots were generated in OriginPro. The raw data used to estimate the IBRv2 value is available in the Supplemental Table 1.

### Statistical analysis

Data from each tissue were evaluated for homogeneity of variances and normality of residuals using Levene’s and Shapiro–Wilk tests, respectively. When the assumptions of analysis of variance (ANOVA) were not met, the data were Box-Cox transformed and reassessed for compliance with these assumptions. Subsequently, the data were analyzed by one-way ANOVA for each tissue, followed by Tukey’s post hoc test, adopting a significance level of 5%. Results were expressed as mean ± standard deviation. Statistical analyses were performed using Minitab® version 22, and graphs were generated using GraphPad Prism version 10.

## Results

### Enzymatic responses

**Fig. 1:**
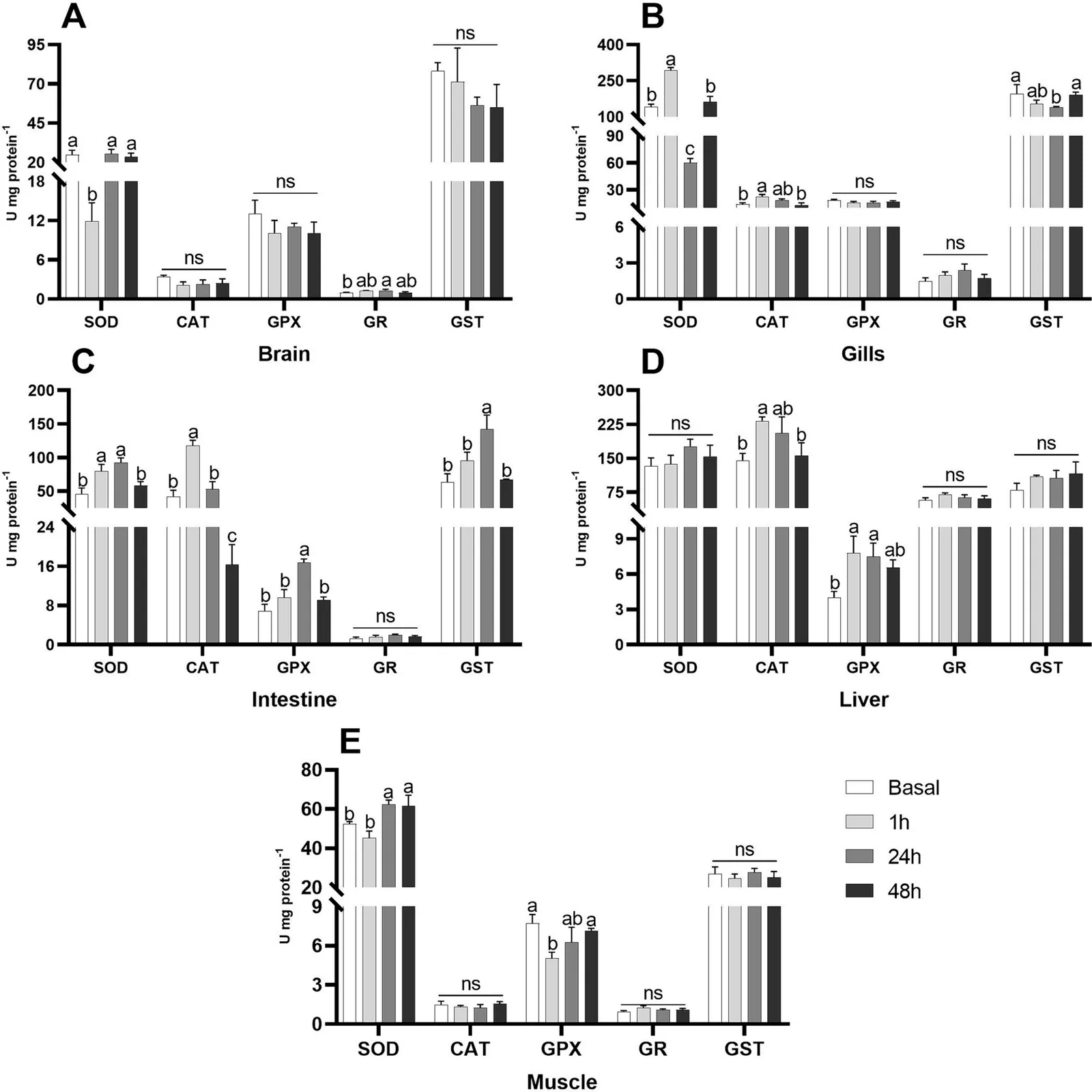
Antioxidant enzymatic responses in different organs and tissues of juvenile tambaqui (*Colossoma macropomum*) exposed to heat shock at 34 °C for 24 h. Samples were collected before temperature elevation (baseline) and at 1, 24, and 48 h after returning to 28 °C (acclimation/control temperature). Panels show the activity of superoxide dismutase (SOD), catalase (CAT), glutathione peroxidase (GPx), glutathione reductase (GR), and glutathione S-transferase (GST) over time in the brain (**A**), gills (**B**), intestine (**C**), liver (**D**), and muscle (**E**). Data are presented as mean ± SD; tank means were used for ANOVA (statistical n = 3 tank means per time point; 18 fish sampled per time point). Different lowercase letters indicate significant differences among sampling times within the same tissue according to Tukey’s test (p < 0.05); ns indicates no significant difference

No mortality was observed during the experimental period. The results of antioxidant enzymes in juvenile *C*. *macropomum* are presented in Fig. 1 (A–E) and indicate time-dependent variation in enzymatic activity within each analyzed tissue.

In the brain (Fig. 1A), there was a significant effect of time on SOD (F = 15.09; p = 0.001) and GR (F = 5.26; p = 0.027). SOD activity was reduced at 1 h compared to the other time points (baseline, 24 h, and 48 h). For GR, higher activity was observed at 24 h relative to baseline, while 1 h and 48 h showed intermediate values. CAT (F = 3.25; p = 0.081), GPX (F = 2.12; p = 0.176), and GST (F = 2.09; p = 0.180) activities did not vary significantly over time.

In the gills (Fig. 1B), time significantly influenced SOD (F = 128.12; p < 0.001), CAT (F = 11.50; p = 0.003), and GST (F = 5.24; p = 0.023) activities. SOD increased at 1 h and decreased at 24 h, with recovery at 48 h to levels similar to baseline. CAT activity was higher at 1 h, with no difference between baseline and 48 h. GST decreased at 24 h compared to baseline and 48 h. GPX (F = 2.95; p = 0.098) and GR (F = 3.26; p = 0.081) activities did not differ significantly over time.

In the intestine (Fig. 1C), a significant effect of time was observed for SOD (F = 19.83; p < 0.001), CAT (F = 76.28; p < 0.001), GPX (F = 20.27; p < 0.001), and GST (F = 20.49; p < 0.001). SOD activity was higher at 1 h and 24 h compared to baseline and 48 h. CAT peaked at 1 h and markedly decreased at 48 h. GPX and GST increased at 24 h and remained lower at the other time points. GR activity (F = 3.25; p = 0.081) did not vary significantly over time.

In the liver (Fig. 1D), temporal variation was observed in CAT (F = 8.50; p = 0.007) and GPX (F = 8.85; p = 0.006). CAT activity increased at 1 h, showed intermediate values at 24 h, and returned at 48 h to levels similar to baseline. GPX activity was higher at 1 h and 24 h compared to baseline, remaining at intermediate levels at 48 h. SOD (F = 2.95; p = 0.098), GR (F = 2.63; p = 0.122), and GST (F = 2.54; p = 0.130) did not differ significantly over time.

In the muscle (Fig. 1E), time significantly affected SOD (F = 16.19; p = 0.001) and GPX (F = 8.05; p = 0.008). SOD activity was higher at 24 h and 48 h compared to baseline and 1 h. GPX activity decreased at 1 h, with recovery at 48 h and intermediate values at 24 h. CAT (F = 1.26; p = 0.352), GR (F = 3.94; p = 0.054), and GST (F = 0.83; p = 0.515) showed no significant differences over time.

### Oxidative damage markers

**Fig. 2:**
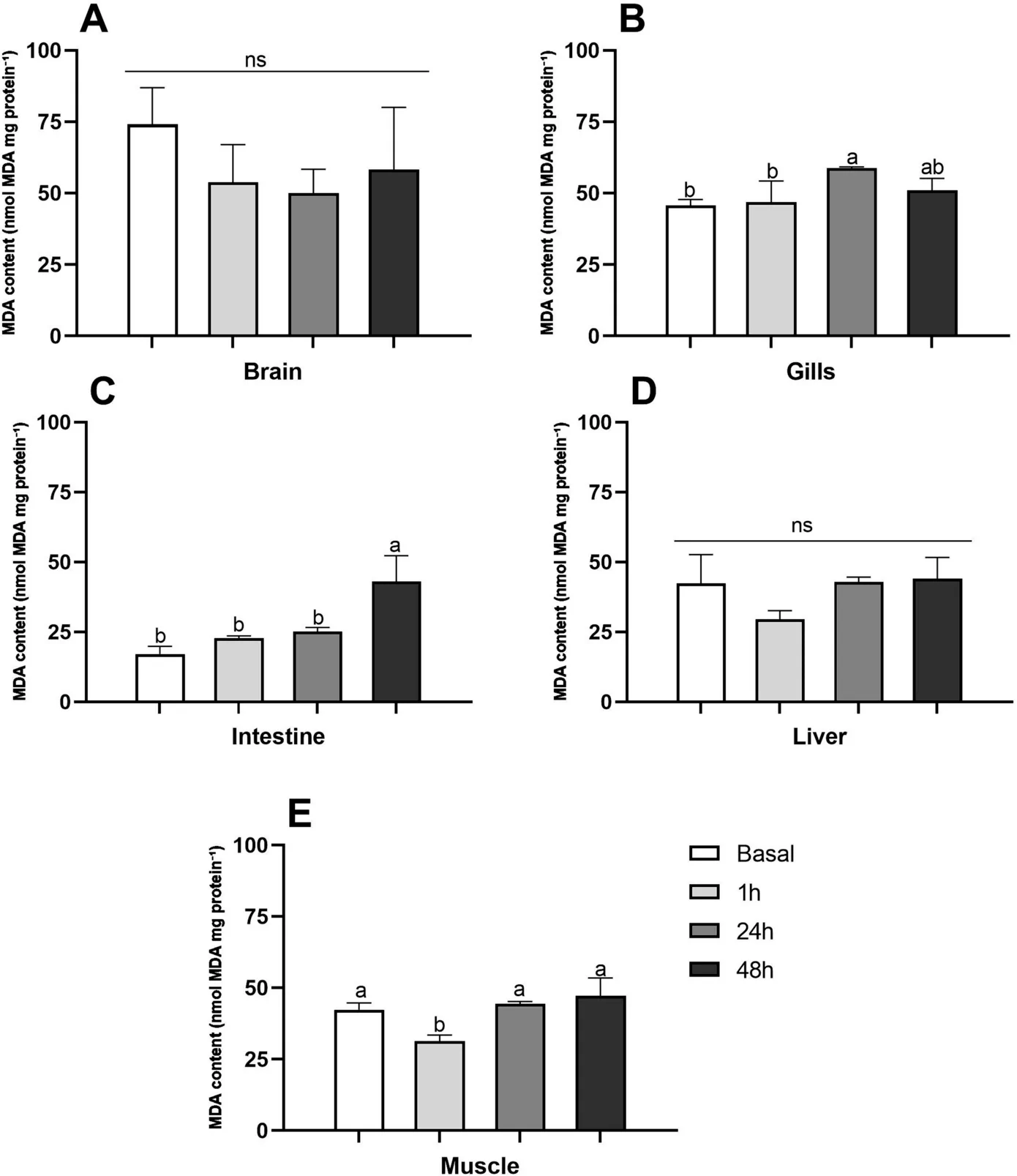
Malondialdehyde (MDA) content, determined by the thiobarbituric acid reactive substances (TBARS) assay, in different organs and tissues of juvenile tambaqui (*Colossoma macropomum*) exposed to heat shock at 34 °C for 24 h. Samples were collected before temperature elevation (baseline) and at 1, 24, and 48 h after returning to 28 °C (acclimation/control temperature). Panels show lipid peroxidation (LPO), measured as MDA levels, over time in the brain (**A**), gills (**B**), intestine (**C**), liver (**D**), and muscle (**E**). Data are presented as mean ± SD; tank means were used for ANOVA (statistical n = 3 tank means per time point; 18 fish sampled per time point). Different lowercase letters indicate significant differences among sampling times within the same tissue according to Tukey’s test (p < 0.05); ns indicates no significant difference

The results for MDA content are presented in Fig. 2 (A–E) and indicate significant temporal variation in each tissue. In the brain (Fig. 2A) and liver (Fig. 2D), MDA levels (F = 1.52; p = 0.282) and (F = 3.26; p = 0.081), respectively, did not differ significantly over time. In contrast, in the gills (Fig. 2B), MDA (F = 5.46; p = 0.024) increased at 24 h compared to baseline and 1 h, while at 48 h it showed intermediate values. In the intestine (Fig. 2C), MDA (F = 13.95; p = 0.002) was higher at 48 h compared to the other time points. In the muscle (Fig. 2E), MDA (F = 11.49; p = 0.003) decreased at 1 h relative to baseline, 24 h, and 48 h, which showed similar values to each other.

**Fig. 3:**
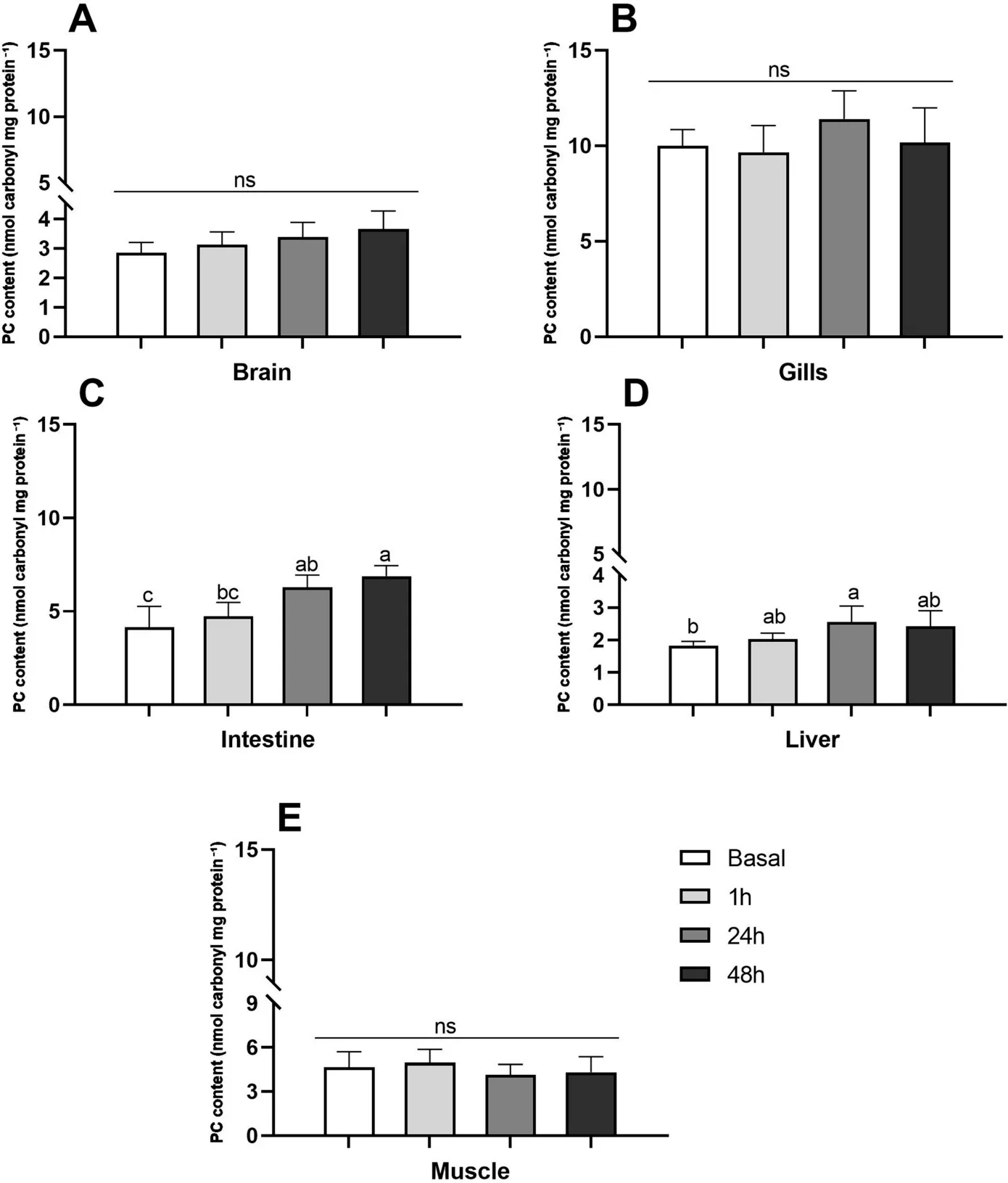
Protein carbonyl (PC) content, in different organs and tissues of juvenile tambaqui (*Colossoma macropomum*) exposed to heat shock at 34 °C for 24 h. Samples were collected before temperature elevation (baseline) and at 1, 24, and 48 h after returning to 28 °C (acclimation/control temperature). Panels show PC levels over time in the brain (**A**), gills (**B**), intestine (**C**), liver (**D**), and muscle (**E**). Data are presented as mean ± SD; tank means were used for ANOVA (statistical n = 3 tank means per time point; 18 fish sampled per time point). Different lowercase letters indicate significant differences among sampling times within the same tissue according to Tukey’s test (p < 0.05); ns indicates no significant difference

The results for protein carbonyl content are presented in Fig. 3 (A–E) and indicate time-dependent variation among tissues. No significant differences were observed over time in the brain (Fig. 3A; F = 1.52; p = 0.283), gills (Fig. 3B; F = 0.84; p = 0.507), or muscle (Fig. 3E; F = 0.47; p = 0.709). In the liver (Fig. 3D), values (F = 4.49; p = 0.041) were higher at 24 h compared to baseline and 1 h, while 48 h showed intermediate levels. In the intestine (Fig. 3C), levels (F = 7.67; p = 0.010) increased over time, with higher values at 24 h and 48 h, reaching the highest levels at 48 h.

### Integrated Biomarker Response (IBRv2)

**Fig. 4:**
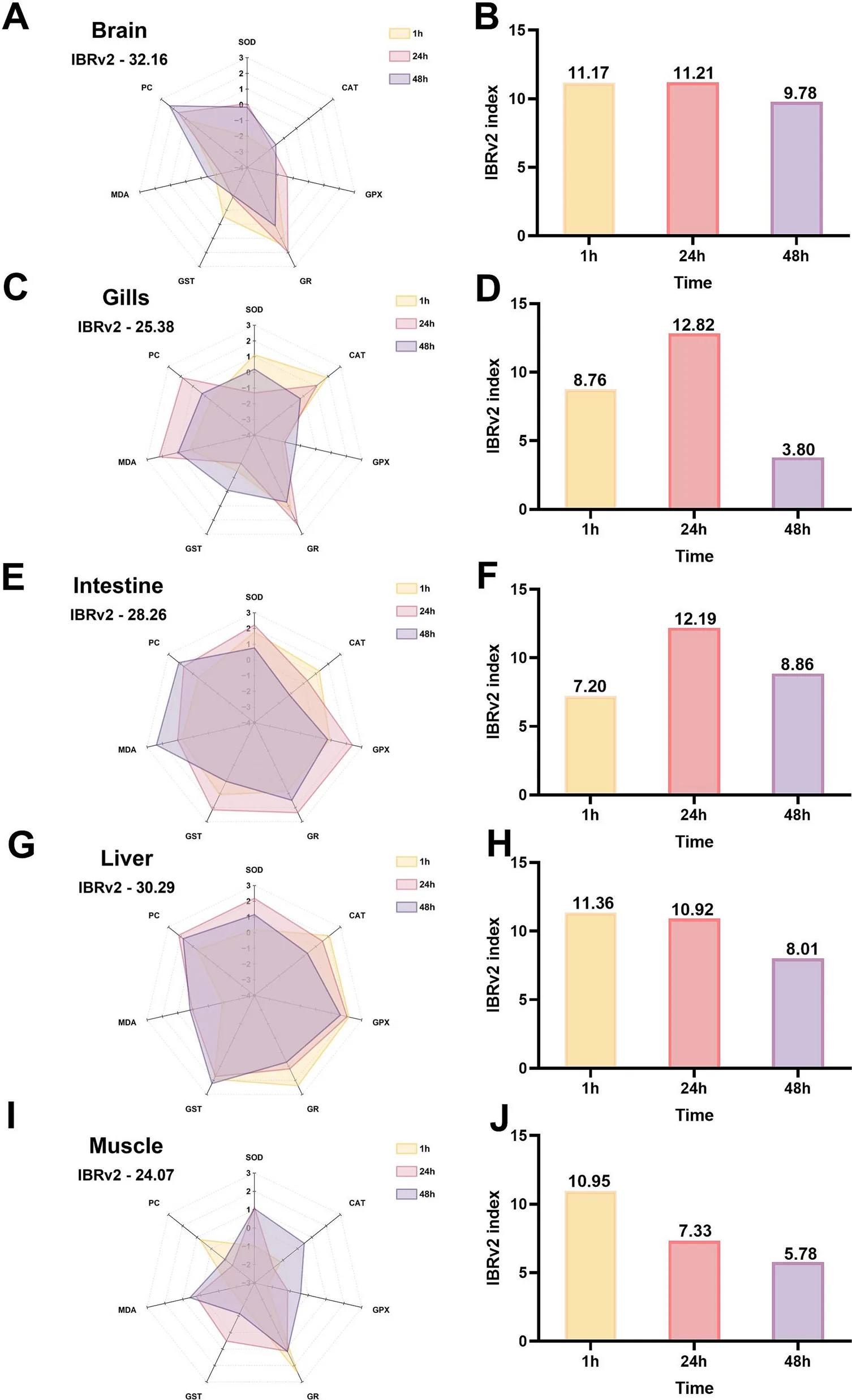
Integrated Biomarker Response (IBRv2) in different organs and tissues of juvenile tambaqui (*Colossoma macropomum*) exposed to heat shock at 34 °C for 24 h. Samples were collected before temperature elevation (baseline) and at 1, 24, and 48 h after returning to 28 °C (acclimation/control temperature). Radar plots illustrate the total IBRv2 and the IBRv2 index over time in the brain (**A, B**), gills (**C, D**), intestine (**E, F**), liver (**G, H**), and muscle (**I, J**), respectively. IBRv2 was calculated from tank-level means (n = 3 tank means per time point; 18 fish sampled per time point)

The Integrated Biomarker Response (IBRv2) revealed clear tissue- and time-dependent patterns (Fig. 4A–J). Considering the total IBRv2 per tissue, the following descriptive ranking was observed in descending order: brain (IBRv2 = 32.16) > liver (IBRv2 = 30.29) > intestine (IBRv2 = 28.26) > gills (IBRv2 = 25.38) > muscle (IBRv2 = 24.07) (Fig. 4A, C, E, G, and I), respectively.

The IBRv2 index over time (Fig. 4B, D, F, H, and J) showed distinct temporal profiles for each tissue. In the brain, values were similar at 1 h (11.17) and 24 h (11.21), decreasing at 48 h (9.78) (Fig. 4B). In the gills, the index increased from 1 h (8.76) to 24 h (12.82), followed by a marked decline at 48 h (3.80) (Fig. 4D). In the intestine, the lowest value occurred at 1 h (7.20), peaking at 24 h (12.19) and decreasing at 48 h (8.86) (Fig. 4F). In the liver, the highest values were observed at 1 h (11.36) and 24 h (10.92), with a reduction at 48 h (8.01) (Fig. 4H). In the muscle, the IBRv2 index was highest at 1 h (10.95) and progressively decreased at 24 h (7.33) and 48 h (5.78) (Fig. 4J).

## Discussion

*Colossoma macropomum* has an optimal temperature range for growth and reproduction between 25 and 32 °C, whereas temperatures above 35 °C may impair physiological performance (Amanajás et al. 2024). In the present study, fish were exposed to 34 °C for 24 h to simulate heatwave conditions that are increasingly affecting Amazonian ecosystems (Mugwanya et al. 2022; Sadayappan and Li 2025). This temperature represents an ecologically relevant sublethal threshold for evaluating heat stress responses in this species (Amanajás et al. 2024). Our results demonstrate that 48 h after returning to the control temperature, antioxidant enzyme activities had largely returned to baseline levels in the brain, gills, liver, intestine (except CAT), and muscle (except SOD). Regarding tissue damage markers (MDA and PC), the intestine was the most affected organ. Overall, gills and muscle showed the smallest deviation from baseline after 48 h, indicating a greater capacity for recovery compared to the other tissues.

Previous studies have shown that environmental stressors such as fasting (Melo et al. 2024a), hypoxia (Mattioli et al. 2025), contaminants (Patel et al. 2022), and heat stress (da Costa et al. 2021; Amanajás et al. 2024) induce distinct antioxidant responses in organs and tissues of fish. Among these stressors, elevated temperature is associated with oxidative stress by increasing metabolic rate and oxygen consumption, enhancing mitochondrial electron leakage, and stimulating non-mitochondrial ROS sources such as oxygenases (e.g., cytochrome P_450_), NADPH oxidases, and peroxisomal enzymes, while potentially overwhelming antioxidant defenses (Sáez-Arteaga et al. 2024; Ma et al. 2025) Although several studies have demonstrated the effects of heat stress on the antioxidant capacity of tambaqui, most have focused on a limited number of organs and have not evaluated the species’ ability to recover, which remains poorly understood (Castro et al. 2020; dos Santos Silva et al. 2024).

Given the tissue-specific patterns observed, we examined each organ individually. In the brain, antioxidant enzyme activities were fully restored 48 h after heat stress, returning to baseline levels. The fish brain is a metabolically active organ with high susceptibility to oxidative damage due to its elevated content of polyunsaturated fatty acids and membrane-rich lipids (Hong et al. 2014). In *Astyanax lacustris* exposed to elevated temperature, activation of glutathione-related enzymes and progressive thermal acclimation were reported, along with increased glucose-6-phosphate dehydrogenase (G6PDH) activity at 31 °C (Ratko et al. 2022). G6PDH catalyzes the rate-limiting step of the pentose phosphate pathway, generating NADPH (Guillen et al. 2019). NADPH serves as a reducing equivalent for glutathione reductase (GR), enabling the regeneration of reduced glutathione (GSH) from oxidized glutathione (GSSG). Thus, enhanced G6PDH activity supports GR function and maintenance of redox homeostasis (Matviishyn et al. 2014). Our findings directly align with this mechanism: the transient increase in GR and concurrent decrease in SOD suggest a rapid metabolic shift toward this glutathione-dependent defense. Because this pathway was effectively mobilized, MDA and PC levels remained not significantly altered, demonstrating that the brain successfully prevented structural oxidative damage under thermal challenge (Wu et al. 2015; Ratko et al. 2022).

In addition to the brain, the gills are highly metabolically active, as they sustain continuous gas exchange and osmoregulatory processes (Islam et al. 2020). Due to their direct contact with the external environment, they are often primary targets of stressors such as elevated temperature. Modulation of antioxidant enzyme expression and activity, along with shifts in metabolic rate, represents one of the main mechanisms involved in coping with heat shock (Islam et al. 2022). In our study, the first-line antioxidant enzymes SOD and CAT, together with the detoxification enzyme GST, showed altered activities at 1 h and 24 h following heat shock, but returned to baseline levels after 48 h. Similar to our results, Pikeperch (*Sander lucioperca*) heat-stressed at 29 °C for up to 48 h increased the activities of SOD and CAT during the early period of heat stress, but decreased them in the later period. Additionally, transcriptomic profiles in gills revealed alterations in the transcript levels of genes related to the antioxidant system, heat shock proteins, and energy metabolism. These authors argue that the early increase in SOD and CAT activity reflects the activation of antioxidant defenses during the initial phase of heat stress. However, when ROS production exceeds the neutralizing capacity of this system, oxidative damage may ensue, potentially impairing enzyme function through lipid peroxidation (Chen et al. 2020). Indeed, in our study, the levels of MDA and PC increased at 1 h and 24 h post-recovery, but returned to intermediate levels at 48 h. Together, these results indicate that acute heat stress may have overwhelmed the antioxidant defenses in the gills, resulting not only in transient oxidative imbalance but also in lipid peroxidation and potential structural disturbances in the tissue. Such responses are consistent with reports of morphological and histological alterations in gill tissue under elevated temperature conditions (Yan et al. 2022; Abdel-Tawwab et al. 2025; Liang et al. 2025).

The intestine also represents a highly sensitive organ to thermal challenge. As a multifunctional tissue responsible for nutrient absorption, immune surveillance, and maintenance of the intestinal microbiota, it plays a central role in overall physiological homeostasis (He et al. 2019, 2021). Heat stress has been shown to compromise intestinal integrity by disrupting epithelial barriers, altering antioxidant defenses, and promoting shifts in microbial composition, which may collectively exacerbate oxidative and inflammatory processes (Zheng et al. 2019; Lian et al. 2020; Elbahnaswy et al. 2024). In our study, we observed increased activities of nearly all evaluated enzymes (SOD, CAT, GPx, and GST) during the early period following heat shock. However, their activities declined over time and largely returned to baseline levels after 48 h of recovery. In contrast, MDA and PC levels did not normalize, remaining elevated even after 48 h, suggesting persistent oxidative damage despite the apparent restoration of enzymatic antioxidant defenses.

The lack of immediate damage resolution is hypothesized to occur because thermal stress disrupts intestinal tight junctions, potentially causing a "leaky gut" (Zhou et al. 2022). This barrier failure may allow luminal endotoxins and altered microbiota by products to translocate and trigger secondary localized inflammation, which may contribute to sustained lipid peroxidation (MDA) independently of the initial thermal shock (Li et al. 2023; Fang et al. 2023). Furthermore, while antioxidant enzymes can rapidly scavenge newly formed ROS, the clearance of persistently accumulated carbonylated proteins (PC) may involve slower downstream cellular repair processes, such as the unfolded protein response (UPR) and tissue remodeling. Thus, recovery of antioxidant enzyme activity may precede the resolution of structural damage. However, these proposed mechanisms were not experimentally evaluated in the present study. Nevertheless, our findings are consistent with previous studies showing activation of antioxidant defense mechanisms and tissue damage following heat stress (Zhu et al. 2022; Zhao et al. 2024; Zhai et al. 2025). For instance, Rainbow trout (*Oncorhynchus mykiss*) kept at high temperatures of 18 and 21^◦^C for up to 14 days showed histopathological damage in the intestine, alterations in the expression of pro-inflammatory cytokines, and decreased microbial diversity compared to the control group kept at 15^◦^C (Zhao et al. 2024). Interestingly, previous studies have shown that dietary feed additives, such as probiotics, prebiotics, and antioxidant compounds, may mitigate heat-stress-related intestinal alterations (Li et al. 2021, 2023; Dawood et al. 2022). This represents a promising strategy for improving resilience and maintaining productivity in fish farming under elevated temperature conditions.

The liver is also a key target in thermal stress studies, as it plays a central role in metabolic regulation, detoxification, and maintenance of redox balance (Sáez-Arteaga et al. 2024). In our study, CAT and GPx activities increased at 1 and 24 h following heat shock but returned to baseline levels after 48 h of recovery, whereas the activities of the other enzymes remained unchanged. Likewise, no significant changes were observed in PC and MDA levels, indicating that oxidative damage was effectively prevented in this tissue. Previous studies have demonstrated detrimental hepatic effects under prolonged thermal stress (Roychowdhury et al. 2020, 2021; Khieokhajonkhet et al. 2023). For instance, goldfish (*Carassius auratus*) acclimated at 34^◦^C for 10 weeks showed increased activity of hepatic enzymes ALT and AST, higher glucose and cortisol levels, as well as swollen hepatocytes, nuclei displacement, and infiltration of inflammation, as revealed by the histopathological analysis (Khieokhajonkhet et al. 2023). However, investigations into post-stress recovery remain scarce. A multi-omics study of juvenile mandarin fish (*Siniperca chuatsi*) exposed to elevated temperatures, followed by a recovery period, revealed partial metabolic restoration in the liver, including reactivation of immune pathways, cytoskeletal remodeling, and recovery of key metabolic processes such as glycolysis and *β*-oxidation (Guo et al. 2025). Together, these findings highlight the adaptive potential of freshwater fish under warming scenarios and suggest that the liver exhibits considerable metabolic plasticity, initiating timely compensatory adjustments to restore (at least partially) its metabolic capacity and mitigate the risk of permanent structural damage following acute thermal stress.

Building on the metabolic plasticity observed in the liver, which can rapidly adjust its metabolism to cope with thermal challenges, other tissues, such as skeletal muscle, also exhibit adaptive responses to temperature fluctuations. Skeletal muscle constitutes 40–60% of body mass and plays a central role in somatic growth and locomotion, undergoing molecular adjustments under thermal stress (Madeira et al. 2017). In addition to its structural function, muscle metabolism is influenced by endocrine pathways such as growth hormone (GH) and insulin-like growth factor (IGF), which regulate myogenesis and energy allocation (Sáez-Arteaga et al. 2024). In our study, skeletal muscle showed increased SOD activity even after 48 h of recovery, while GPx and GR activities were transiently altered but returned to baseline levels. This uncoupled enzymatic response creates a stoichiometric imbalance in the antioxidant defense system, theoretically leading to intracellular hydrogen peroxide (H_2_O_2_) accumulation, which acts as a prooxidant agent. However, despite this prooxidant risk, MDA levels decreased at 1 h and remained unchanged thereafter, whereas PC levels were not affected.

The effective mitigation of H_2_O_2_ toxicity, despite unchanged CAT and GPx responses, may be explained by the activation of alternative antioxidant pathways. For instance, the thioredoxin (Trx) and peroxiredoxin (Prx) systems are highly effective in decomposing H_2_O_2_ and preventing oxidative damage in fish tissues when primary peroxidases are not upregulated (Tolomeo et al. 2016; Chu et al. 2019; Li et al. 2022)**.** Together, these results suggest that muscle tissue mounts a coordinated antioxidant response. In this context, a controlled response is defined as a targeted and energetically efficient strategy: rather than mounting a generalized and metabolically costly upregulation of all primary antioxidant enzymes (Jiménez and Nash-Braun 2023), the tissue selectively relies on specific alternative pathways to precisely neutralize ROS. This allows the muscle to maintain cellular homeostasis during thermal challenge without compromising its overall energy allocation. However, because the expression or activity of these alternative antioxidant systems was not evaluated in the present study, their contribution remains to be confirmed.

The IBRv2 allowed a clear visualization of tissue-specific oxidative responses to heat stress. Overall, higher cumulative responses were observed in the brain and liver, followed by the intestine, gills, and muscle, suggesting that these tissues were more responsive to the thermal challenge. Biologically, this high responsiveness in the liver and brain is closely linked to their intense metabolic rates, continuous oxygen consumption, and central roles in systemic homeostasis and detoxification. Such characteristics inherently increase ROS production during thermal stress, demanding a massive and immediate mobilization of antioxidant defenses to prevent structural damage (Tseng et al. 2011; Rossi et al. 2017; Madeira et al. 2017). Temporal patterns also differed among tissues, with early responses in the brain, liver, and muscle, whereas intestine and gills showed a peak at 24 h. Notably, gills and muscle exhibited the smallest deviation from baseline levels after 48 h, indicating a more complete recovery of redox homeostasis compared with the other tissues.

This faster restoration and lower cumulative deviation reflect the metabolic plasticity of these tissues. Instead of a metabolically costly overactivation of primary enzymes, the muscle and gills likely rely on alternative detoxification pathways, barrier adaptations, or anaerobic metabolic shifts, enabling a highly efficient resolution of the oxidative challenge (Jiménez and Nash-Braun 2023; Pereira et al. 2023). Furthermore, other protective mechanisms, such as heat shock proteins (HSPs), may also contribute to the maintenance of cellular homeostasis under thermal stress (Oksala et al. 2014). However, these pathways were not assessed in the present study, representing a limitation of our work. Finally, it is also important to note that the absence of a time-matched normothermal control group is a limitation of this study. Thus, future studies should include it to distinguish heat-stress effects from handling, fasting, sampling time, or background temporal variation.

## Conclusion

In conclusion, tissues such as gills and muscle showed a faster return to baseline conditions, indicating a more efficient recovery. The intestine, on the other hand, displayed more persistent alterations, particularly in oxidative damage markers. From an applied aquaculture perspective, this delayed intestinal recovery signifies that nutrient absorption and mucosal immune barriers remain severely compromised even after water temperatures normalize. Consequently, animal welfare may be threatened by a higher risk of secondary infections and reduced growth performance during the recovery phase. These findings suggest that future studies should evaluate whether temporary feed restriction could reduce the metabolic load on the damaged gut during recovery from heat stress. Likewise, future studies should investigate whether functional feed additives, such as dietary antioxidants or probiotics, may accelerate intestinal repair before resuming standard feeding regimes. Moreover, our findings highlight that the recovery from heat stress in *C*. *macropomum* is not uniform among tissues, emphasizing the importance of multi-tissue approaches when assessing physiological resilience. Future studies should investigate additional mediators beyond oxidative stress, including inflammatory and metabolic pathways, as well as evaluate longer recovery periods to better understand the full scope of physiological adjustments following thermal challenges.

## Supplementary Information

Below is the link to the electronic supplementary material.

Supplementary file1 (XLSX 36 KB)

## Data Availability

The data that support the findings of this study are available on request from the corresponding author, LDSM.
